# Mitochondria transfer from mesenchymal stem cells structurally and functionally repairs renal proximal tubular epithelial cells in diabetic nephropathy *in vivo*

**DOI:** 10.1038/s41598-019-40163-y

**Published:** 2019-03-26

**Authors:** Naoto Konari, Kanna Nagaishi, Shin Kikuchi, Mineko Fujimiya

**Affiliations:** 1 Second Department of Anatomy, Sapporo Medical University, Sapporo, 060-8556 Japan; 2 First Department of Anatomy, Sapporo Medical University, Sapporo, 060-8556 Japan

## Abstract

The underlying therapeutic mechanism of renal tubular epithelium repair of diabetic nephropathy (DN) by bone marrow-derived mesenchymal stem cells (BM-MSCs) has not been fully elucidated. Recently, mitochondria (Mt) transfer was reported as a novel action of BM-MSCs to rescue injured cells. We investigated Mt transfer from systemically administered BM-MSCs to renal proximal tubular epithelial cells (PTECs) in streptozotocin (STZ)-induced diabetic animals. BM-MSCs also transferred their Mt to impaired PTECs when co-cultured *in vitro*, which suppressed apoptosis of impaired PTECs. Additionally, BM-MSC-derived isolated Mt enhanced the expression of mitochondrial superoxide dismutase 2 and Bcl-2 expression and inhibited reactive oxygen species (ROS) production *in vitro*. Isolated Mt also inhibited nuclear translocation of PGC-1α and restored the expression of megalin and SGLT2 under high glucose condition (HG) in PTECs. Moreover, isolated Mt directly injected under the renal capsule of STZ rats improved the cellular morphology of STZ-PTECs, and the structure of the tubular basement membrane and brush border *in vivo*. This study is the first to show Mt transfer from systemically administered BM-MSCs to damaged PTECs *in vivo*, and the first to investigate mechanisms underlying the potential therapeutic effects of Mt transfer from BM-MSCs in DN.

## Introduction

Mitochondria (Mt) transfer has been proposed as a novel action of mesenchymal stem cells (MSCs). Previous studies demonstrated the phenomenon of Mt transfer from MSCs in several specific organs *in vivo* and *in vitro*^1–5^. Bone marrow-derived MSCs (BM-MSCs) transferred their Mt to lung epithelial cells in an airway injury model^1^, which increased adenosine triphosphate (ATP) levels in airway epithelial cells. BM-MSCs transferred their Mt to cardiomyocytes and suppressed apoptosis or degeneration in an ischaemia/reperfusion model^6^ and an anthracycline-induced cardiomyopathy model^7^, or reprogrammed differentiated cardiomyocytes to progenitor-like cells^3^. The neuroprotective effects of Mt transfer were also revealed in cortical neurons after stroke^4^. These studies concluded that Mt transfer from MSCs increased activity of the respiratory chain complex and ATP levels in damaged cells *in vivo and in vitro*.

Diabetic nephropathy (DN), one of the most serious causes of chronic kidney disease, has a high mortality rate in patients with diabetes^8^. Mitochondrial dysfunction induced by hyperglycaemia contributes to DN^9^, which results from the overproduction of reactive oxygen species (ROS) and reduced ATP production, especially in renal tubular epithelial cells^10,11^. Proximal tubules perform active transport for the reabsorption and regulation of water, glucose and electrolytes such as sodium ions, which requires a large amount of energy in the form of ATP^12^. Therefore, functional disorder of Mt impairs the homeostasis of body fluids and the regulation of urine composition in DN. Mt degeneration also leads to cytoskeletal alterations, which induce destruction of the brush border, loss of cell-to-cell contacts and enhanced epithelial shedding^13–15^. Morphological improvements of renal tubular epithelial cells by complementing with functional endogenous Mt are necessary for the repair of renal tubules.

MSCs are a practical source for use in regenerative therapy of the kidney. Renal proximal tubular epithelial cells (PTECs) possess strong regenerative ability upon injury or due to shedding by some strong obstacle, such as acute kidney disease^16–18^. Perico *et al*. recently reported that human umbilical cord-derived MSCs up-regulated ATP levels, mitochondrial polarisation and citrate synthase activity, and suppressed the fragmentation of Mt after cisplatin-induced acute tubular epithelial cell damage *in vitro* and *in vivo*^19^. Accordingly, Mt transfer from BM-MSCs to PTECs should be advantageous for the repair of PTECs in DN. However, no previous studies have shown Mt transfer from MSCs to tubular epithelial cells either *in vitro* or *in vivo*.

Therefore, in this study, we first present the phenomenon of Mt transfer *in vivo* and *in vitro*, and then investigated its crucial roles for the repair of damaged PTECs in the kidney of a DN model as follows: (1) anti-apoptotic and anti-degenerative effects for PTECs; (2) inhibition of ROS production by regulating mitochondria-related factors, e.g., SOD2, Bcl-2, Bax and PGC-1α; (3) recovery of the expression of functional transporters in PTECs, e.g., Megalin and SGLT2; and (4) structural improvement of PTECs and reorganisation of tubular epithelium. Our findings provide new insights that describe the mechanisms that MSCs use to exert therapeutic effects against DN.

## Results

### DsRed2-Mt are detected in PTECs of STZ mice that were systemically administered MtDsRed2-MSCs

**Figure 1:**
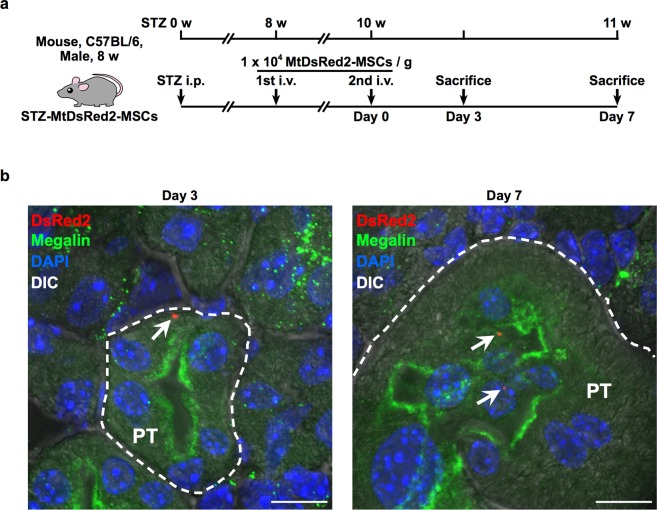
Mt transfer from BM-MSCs to STZ-PTECs in STZ mice. (**a**) Experimental protocol for the administration of BM-MSCs in STZ mice (*n* = 5 per group). (**b**) Immunofluorescence images of megalin (green) expression and localisation of MtDsRed2-MSC-derived Mt (red, white arrows) in proximal tubules of the kidney of STZ mice. Images were obtained 3 and 7 d after systemic administration of MtDsRed2-MSCs. Nuclei are counterstained with DAPI (blue). PT, proximal tubules. Scale bar, 10 µm.

We have established previously a protocol to treat STZ-induced diabetic mice with intravenous administration of rat BM-MSCs; therefore, Mt transfer from BM-MSCs using this model was first demonstrated. To examine whether intravenously injected BM-MSCs transferred their own Mt to PTECs *in vivo*, MtDsRed2-MSCs were administered twice to STZ mice via the tail vein (Fig. 1a). The purpose of this study was to investigate Mt transfer to PTECs *in vivo* as one of the efficacy mechanisms of MSC therapy and not to clarify the therapeutic effect of BM-MSCs only by Mt transfer. Therefore, the objective of this research report was achieved with *n* = 5. DsRed2-Mt were detected in the cytoplasm of PTECs that expressed megalin in STZ mice 3 and 7 d after the last administration of MtDsRed2-MSCs (Fig. 1b). The number of DsRed2-Mt-positive PTECs in each of the five mice was 0.859 ± 0.612 cells/cm^2^ on day 3 and 0.776 ± 0.505 cells/cm^2^ on day 7. Since we sliced the renal tissues with 20 μm thickness in this experiment, the tissue included 0.859 cells/0.002 cm^3^ on day 3 and 0.776 cells/0.002 cm^3^ on day 7. Assuming that the volume of one kidney is 2 cm^3^, the number of DsRed2-Mt positive PTECs per kidney is 859 cells on day 3 and 776 cells on day 7 theoretically.

### MtDsRed2-MSCs transfer endogenous DsRed2-Mt to STZ-PTECs *in vitro*

**Figure 2:**
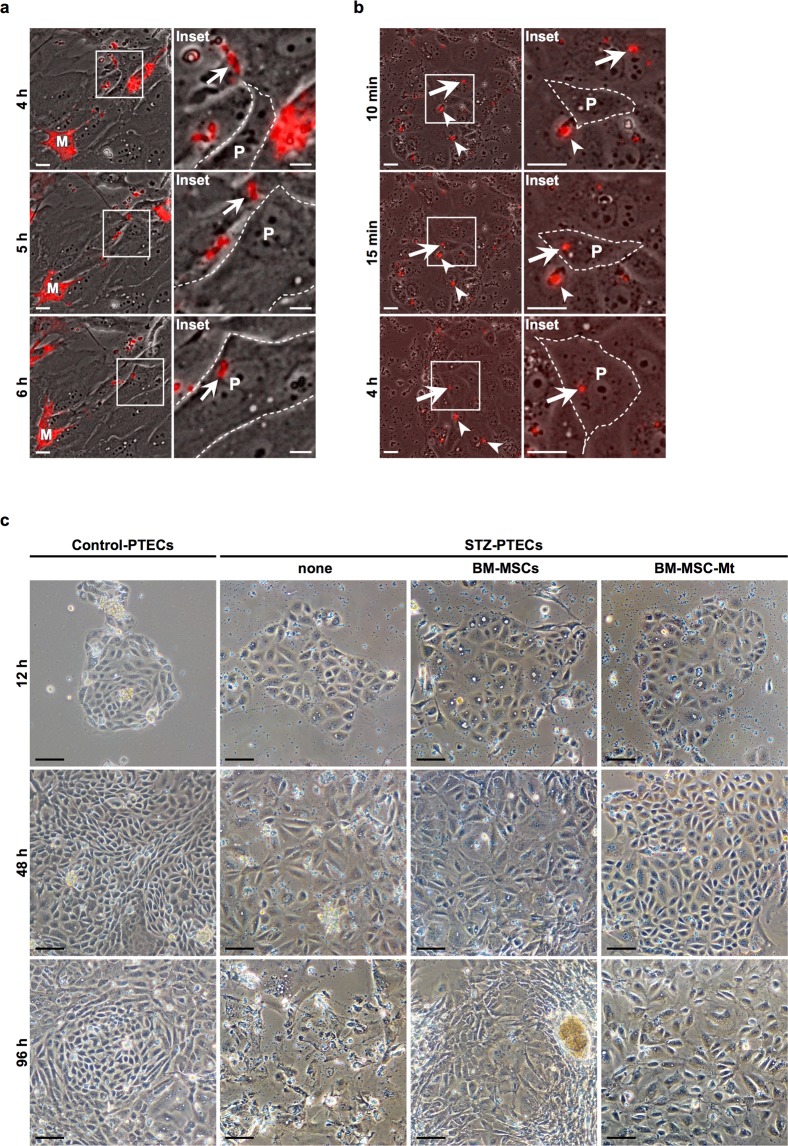
Incorporation of BM-MSC-Mt into STZ-PTECs, and the anti-degenerative effects of BM-MSCs and BM-MSC-Mt *in vitro*. (**a**) Time-lapse images of Mt transfer from MtDsRed2-MSCs to STZ-PTECs. Images were obtained 4, 5 and 6 h after commencing time-lapse observations. Panels on the right show magnified images of the left panels. White arrows track the same DsRed2-Mt. M: MtDsRed2-MSCs, P: PTECs derived from STZ rats (STZ-PTECs). Scale bar, 25 µm. (**b**) Time-lapse images of the incorporation of isolated Mt (BM-MSC-Mt) into STZ-PTECs. Images were obtained at 10 and 15 min and 4 h after commencing time-lapse observations. Panels on the right show magnified images of left panels. White arrows and arrowheads track the same DsRed2-Mt. P: PTECs derived from STZ rats (STZ-PTECs). Scale bar, 25 µm. (**c**) Phase contrast observations of Control-PTECs and STZ-PTECs cultured with or without BM-MSCs and BM-MSC-Mt. Images were obtained 12, 48 and 96 h after commencing the co-culture with BM-MSCs or BM-MSC-Mt. Scale bar, 100 µm.

DsRed2-Mt were transferred into STZ-PTECs when STZ-PTECs were co-cultured with MtDsRed2-MSCs *in vitro* (Fig. 2a; Supplementary Video S1). Some DsRed2-Mt had already transferred into STZ-PTECs 12 h after commencing co-culturing. DsRed2-Mt continued to localise in the cytoplasm of STZ-PTECs for at least 24 h (Supplementary Video S2).

### DsRed2-Mt isolated from MtDsRed2-MSCs are incorporated into STZ-PTECs *in vitro*

DsRed2-Mt from MtDsRed2-MSCs were isolated and added to STZ-PTECs *in vitro* to analyse the Mt-specific actions on STZ-PTECs, excluding other various functions of BM-MSCs. DsRed2-Mt strongly expressed DsRed2 fluorescence and maintained their morphological structure (Supplementary Fig. S2a,b). The biological activity of isolated Mt evaluated by the oxygen consumption rate was maintained (Supplementary Fig. S2c). DsRed2-Mt had already been transferred into STZ-PTECs 12 h after commencing co-culturing (Fig. 2b; Supplementary Video S3). DsRed2-Mt continued to localise in the cytoplasm of STZ-PTECs for at least 24 h (Supplementary Video S4).

### BM-MSCs and Mt isolated from BM-MSCs maintain the colony formation of STZ-PTECs *in vitro*

The influence of BM-MSCs and Mt isolated from BM-MSCs (BM-MSC-Mt) were evaluated in primary cultured STZ-PTECs *in vitro*. According to phase contrast observations, colonies were enlarged for 96 h with normal cell morphology in Control-PTECs (Fig. 2c, leftmost panels), whereas colony growth was retarded and cell integrity absence in STZ-PTECs (Fig. 2c, middle left panels). Colony formation was maintained for 96 h when STZ-PTECs were co-cultured with BM-MSCs (Fig. 2c, middle right panels). When STZ-PTECs were co-cultured with BM-MSC-Mt, colony formation was maintained for 96 h as similar as co-culturing with BM-MSCs (Fig. 2c, rightmost panels).

### MtDsRed2-MSCs and Mt isolated from MtDsRed2-MSCs maintain the expression level of LTL in STZ-PTECs *in vitro*

**Figure 3:**
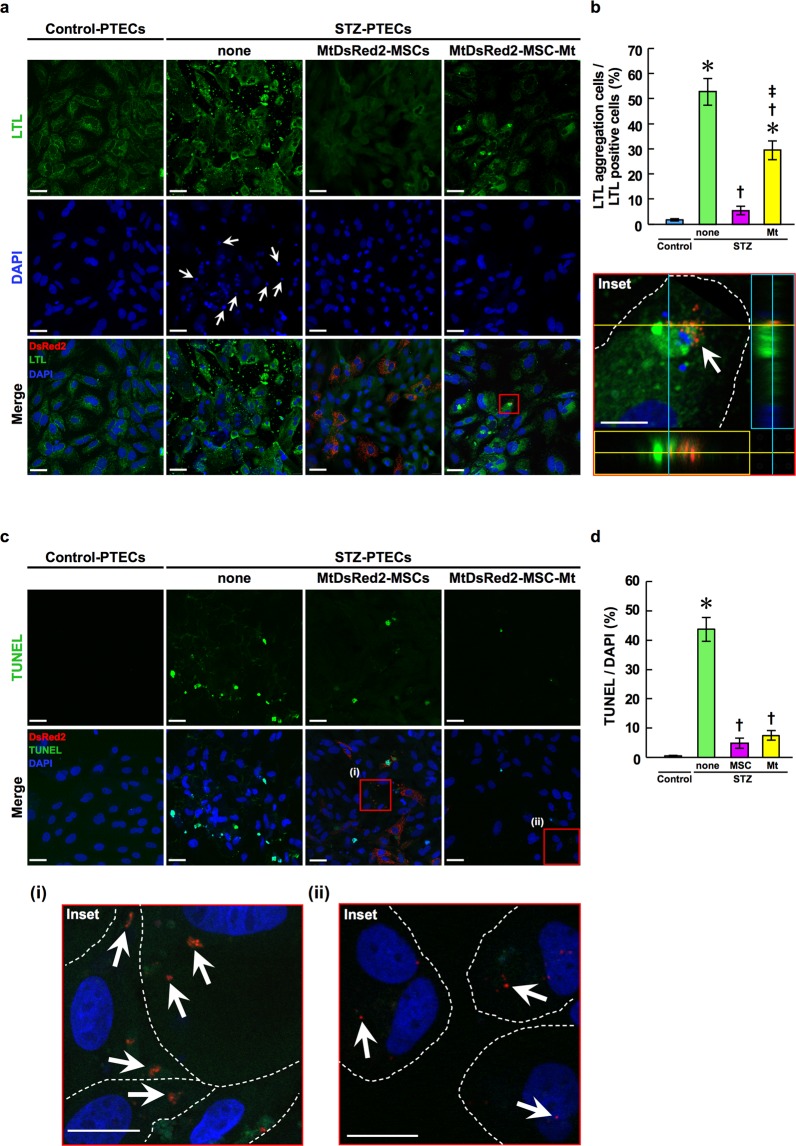
Anti-apoptotic effects of BM-MSCs and BM-MSC-Mt *in vitro*. (**a**) Immunofluorescence images of LTL (green) in Control-PTECs and STZ-PTECs cultured with or without BM-MSCs and BM-MSC-Mt. Images were obtained 96 h after commencing the co-culture with MtDsRed2-MSCs or MtDsRed2-MSC-Mt (red). Nuclei are counterstained with DAPI (blue). White arrows indicate deformed and shrunken nuclei. The white arrows in the insets indicate incorporated BM-MSC-Mt in STZ-PTECs. Experiments were repeated three times independently. Scale bar, 40 μm; 10 µm in insets. (**b**) Quantification of LTL aggregation. Data are shown as mean ± s.e.m. (Control-PTECs, *n* = 6; STZ-PTECs, *n* = 8; STZ-PTECs-MtDsRed2-MSCs, *n* = 4; STZ-PTECs-MtDsRed2-MSC-Mt, *n* = 4). *P < 0.05 Control-PTECs vs. STZ-PTECs, and Control-PTECs vs. STZ-PTECs-MtDsRed2-MSC-Mt; ^†^*P* < 0.05 STZ-PTECs vs. STZ-PTECs-MtDsRed2-MSCs, and STZ-PTECs vs. STZ-PTECs-MtDsRed2-MSC-Mt; ^‡^*P* < 0.05 STZ-PTECs-MtDsRed2-MSCs vs. STZ-PTECs-MtDsRed2-MSC-Mt using ANOVA corrected with the Bonferroni coefficient. (**c**) TUNEL staining (green) of Control-PTECs and STZ-PTECs cultured without or with BM-MSCs and BM-MSC-Mt. Images were obtained 96 h after commencing the co-culture with MtDsRed2-MSCs or MtDsRed2-MSC-Mt (red). Nuclei are counterstained with DAPI (blue). White arrows in insets (i) and (ii) show incorporated DsRed2-Mt in STZ-PTECs. White dotted lines in the insets show the outline of individual STZ-PTECs. Scale bar, 40 μm; 10 µm in insets. Experiments were repeated three times independently. (**d**) Quantification of TUNEL-positive apoptotic cells in Control-PTECs and STZ-PTECs cultured without or with BM-MSCs and BM-MSC-Mt. Data are shown as mean ± s.e.m. (Control-PTECs, *n* = 6; STZ-PTECs, *n* = 10; STZ-PTECs-MtDsRed2-MSCs, *n* = 4; STZ-PTECs-MtDsRed2-MSC-Mt, *n* = 8). *P < 0.05 Control-PTECs vs. STZ-PTECs; ^†^*P* < 0.05 STZ-PTECs vs. STZ-PTECs-MtDsRed2-MSCs, and STZ-PTECs vs. STZ-PTECs-MtDsRed2-MSC-Mt using ANOVA corrected with the Bonferroni coefficient.

Lotus tetragonolobus lectin (LTL), which is a marker of PTECs, was expressed at normal levels in Control-PTECs 96 h after primary culturing (Fig. 3a, leftmost panels and 3b). In contrast, LTL abnormally aggregated in the cytoplasm of STZ-PTECs (Fig. 3a, middle left panels and 3b). Nuclei were deformed and had shrunk in STZ-PTECs when compared with those of Control-PTECs (Fig. 3a, middle left panels and 3b). These abnormalities were reduced by co-culturing with MtDsRed2-MSCs or Mt isolated from MtDsRed2-MSCs (MtDsRed2-MSC-Mt) 96 h after commencing the co-culture (Fig. 3a, middle right panels and rightmost panels and 3b).

### MtDsRed2-MSCs and MtDsRed2-MSC-Mt inhibit apoptosis of STZ-PTECs *in vitro*

The number of terminal deoxynucleotidyl transferase-mediated dUTP nick-end labelling (TUNEL)-positive apoptotic cells increased in STZ-PTECs when compared with that of the Control-PTECs 96 h after primary culturing (Fig. 3c, leftmost panels and middle left panels), whereas the number of apoptotic cells was reduced significantly in STZ-PTECs cultured with MtDsRed2-MSCs 96 h after commencing the co-culture (Fig. 3c, middle right panels and 3d). Notably, TUNEL reactivity was negative in STZ-PTECs that had experienced Mt transfer [Fig. 3c, inset (i)]. The number of apoptotic cells was reduced significantly by the addition of MtDsRed2-MSC-Mt and this number was similar to that observed when co-culturing with MtsRed2-MSCs (Fig. 3c, rightmost panels and 3d). Moreover, TUNEL reactivity was negative in STZ-PTECs that had incorporated DsRed2-Mt into the cytoplasm [Fig. 3c, inset (ii)]. DsRed2-Mt were incorporated into STZ-PTECs in proportion to the amount of isolated Mt added (Supplementary Fig. S3). Additionally, more DsRed2-Mt were incorporated into the cytoplasm of STZ-PTECs than in Control-PTECs at each dose examined (Supplementary Fig. S3).

### BM-MSC-Mt promote the expression of SOD2 and Bcl-2 in endogenous Mt of STZ-PTECs and inhibit ROS production *in vitro*

**Figure 4:**
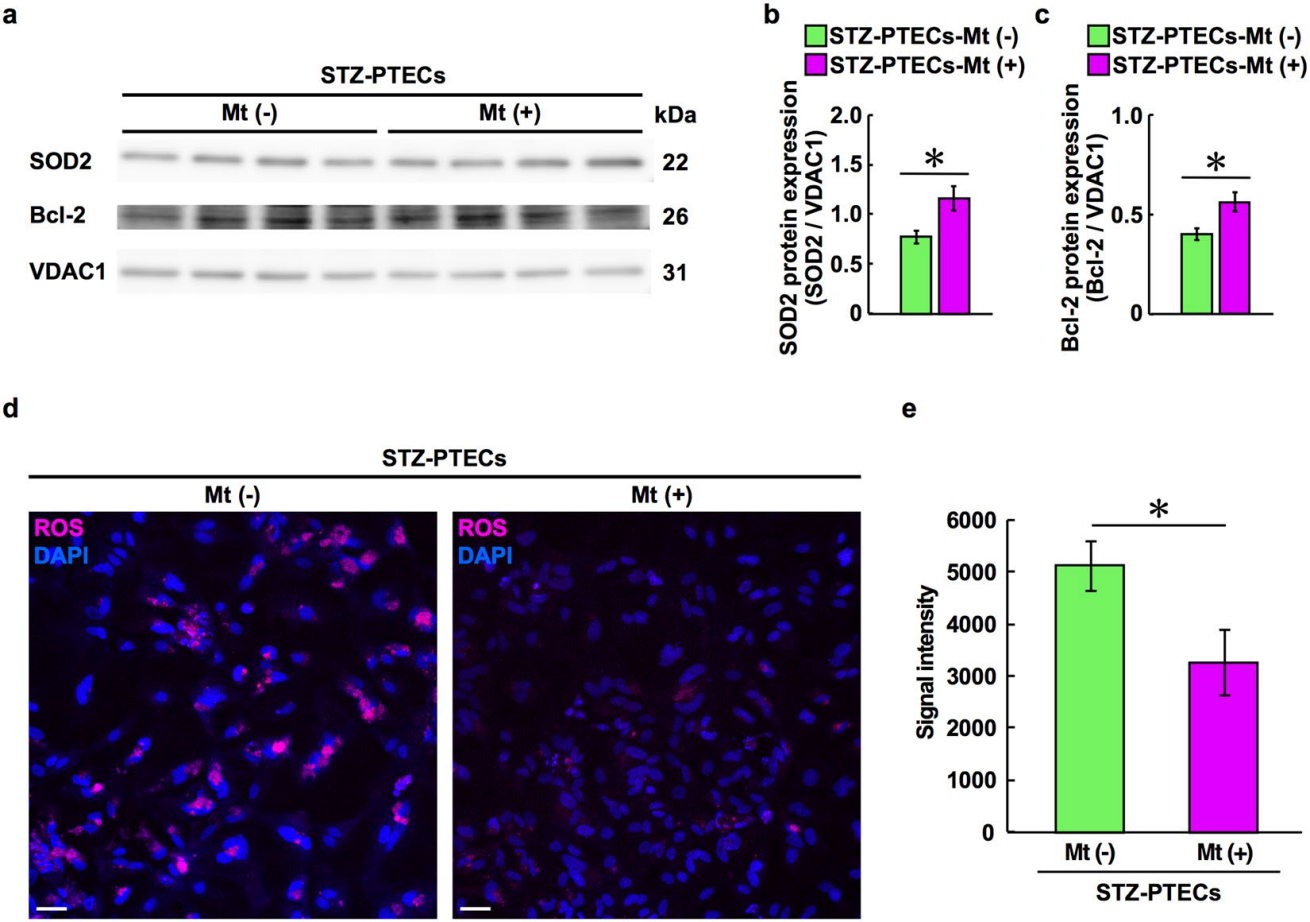
Expression of Mt-related factors and ROS production in STZ-PTECs by the addition of BM-MSC-Mt *in vitro*. (**a**) Expression of SOD2 and Bcl-2 in the Mt-enriched fraction of STZ-PTECs by the addition of BM-MSC-Mt for 24 h (*n* = 4 per group). Cropped images of immunoblots are shown; full-length blots are shown in Supplementary Fig. S7a. (**b**) Densitometric analysis of the expression of SOD2 in (**a**). Data are shown as mean ± s.e.m. (*n* = 4 per group). *P < 0.05 STZ-PTECs-Mt (–) vs. STZ-PTECs-Mt (+) using the unpaired t-test. (**c**) Densitometric analysis of the expression of Bcl-2 in (**a**). Data are shown as mean ± s.e.m. (*n* = 4 per group). *P < 0.05 STZ-PTECs-Mt (–) vs. STZ-PTECs-Mt (+) using the unpaired t-test. (**d**) Fluorescence images of ROS production in STZ-PTECs co-cultured with or without BM-MSC-Mt. Nuclei are counterstained with DAPI (blue). Scale bar, 50 μm. (**e**) Quantification of ROS production in STZ-PTECs by the addition of BM-MSC-Mt. Data are shown as mean ± s.e.m. (*n* = 5 per group). *P < 0.05 STZ-PTECs-Mt (−) vs. STZ-PTECs-Mt (+) using the unpaired t-test.

We analysed the expression of SOD2 and Bcl-2 in STZ-PTECs cultured with or without BM-MSC-Mt to characterise the mechanism of BM-MSC-Mt on STZ-PTECs. SOD2 and Bcl-2 increased significantly in the Mt-enriched fraction of STZ-PTECs cultured with BM-MSC-Mt for 24 h (Fig. 4a–c), whereas ROS production decreased significantly in STZ-PTECs cultured with BM-MSC-Mt *in vitro* (Fig. 4d,e). In contrast, addition of BM-MSC-Mt did not affect the expression of SOD1 in the cytosol-enriched fraction of STZ-PTECs (Supplementary Fig. S4a,b).

### BM-MSC-Mt promote intercellular pathways of PTECs and inhibit ROS production in NRK-52E cells

**Figure 5:**
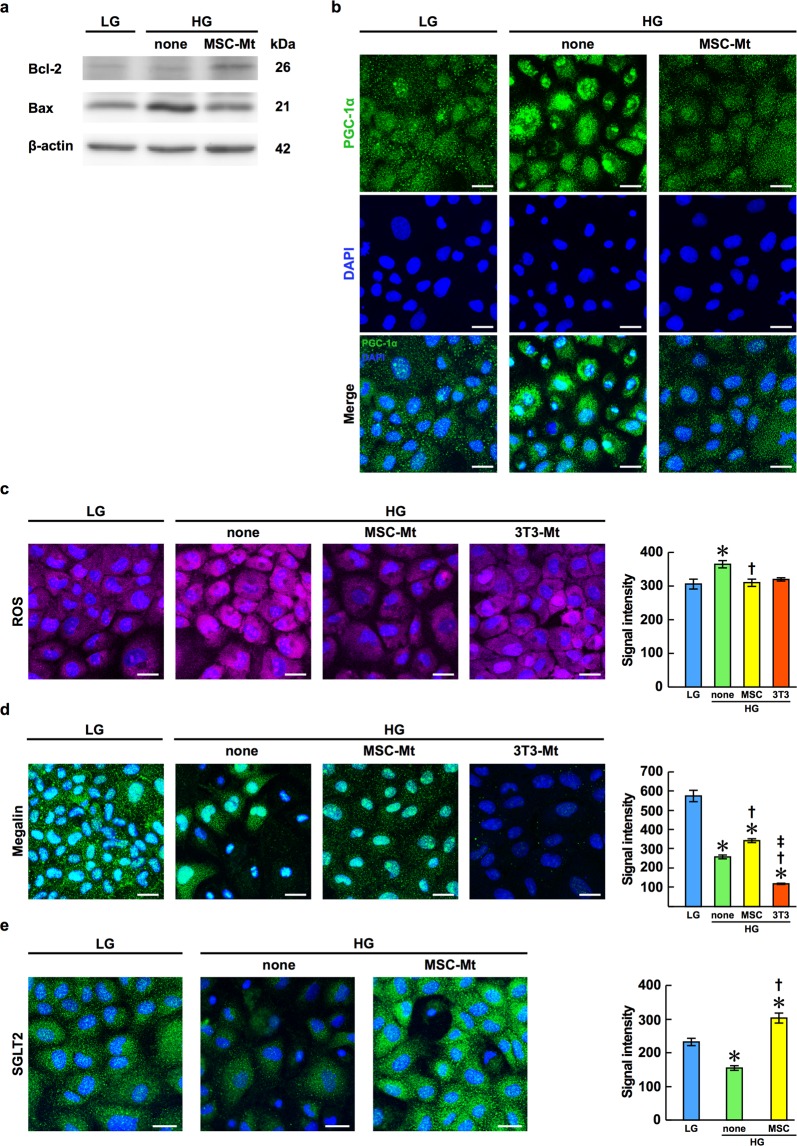
Expression of Mt-related factors, ROS production, and expression of protein and glucose transporters in NRK-52E cells following the addition of BM-MSC-Mt or Mt isolated from NIH-3T3 cells (3T3-Mt). NRK-52E cells were cultured in low glucose (LG) or high glucose (HG) conditions. (**a**) Expression of Bcl-2 and Bax in NRK-52E cells. Cropped images of immunoblots are shown; full-length blots are shown in Supplementary Fig. S7b. (**b**) Immunofluorescence images of PGC-1α (green) in NRK-52E cells. Nuclei are counterstained with DAPI (blue). Scale bar, 25 μm. (**c**) Fluorescence images of ROS production in NRK-52E cells. Nuclei are counterstained with DAPI (blue). Scale bar, 25 μm. Quantification of ROS production is shown in the right panel as the mean ± s.e.m. (*n* = 10 images per group). *P < 0.05 LG vs. HG-none; ^†^*P* < 0.05 HG-none vs. HG-MSC-Mt using ANOVA corrected with the Bonferroni coefficient. (**d**) Immunofluorescence images of megalin (green) in NRK-52E cells. Nuclei are counterstained with DAPI (blue). Scale bar, 25 μm. Quantification of megalin expression is shown in the right panel as the mean ± s.e.m. (*n* = 10 images per group). *P < 0.05 LG vs. HG-none, LG vs. HG-MSC-Mt, and LG vs. HG-3T3-Mt; ^†^*P* < 0.05 HG-none vs. HG-MSC-Mt, and HG-none vs. HG-3T3-Mt; ^‡^*P* < 0.05 HG-MSC-Mt vs. HG-3T3-Mt using ANOVA corrected with the Bonferroni coefficient. (**e**) Immunofluorescence images of SGLT2 (green) in NRK-52E cells. Nuclei are counterstained with DAPI (blue). Scale bar, 25 μm. Quantification of SGLT2 expression is shown in the right panel as the mean ± s.e.m. (*n* = 10 images per group). *P < 0.05 LG vs. HG-none, and LG vs. HG-MSC-Mt; ^†^*P* < 0.05 HG-none vs. HG-MSC-Mt using ANOVA corrected with the Bonferroni coefficient.

We investigated the additional effect of BM-MSC-Mt using NRK-52E cells under both high glucose (HG) and low glucose (LG) conditions to examine the effect of BM-MSC-Mt on intracellular pathways of mitochondrial proteins associated with renal protection. NRK-52E cells are a normal rat kidney proximal tubular epithelial cell line. Bcl-2 expression was suppressed and Bax expression was increased in NRK-52E cells under HG. These changes were corrected by the addition of BM-MSC-Mt (Fig. 5a). Nuclear transition of PGC-1α from the cytoplasm was observed in NRK-52E cells under HG, which was recovered by adding BM-MSC-Mt (Fig. 5b). ROS production increased under HG (Fig. 5c, middle left panel), which decreased significantly when BM-MSC-Mt were added (Fig. 5c, middle right panel). Next, to investigate whether these regulatory effects are specific for Mt derived from BM-MSCs, we isolated Mt from NIH-3T3 cells, which are a mouse fibroblast cell line, and added these Mt to NRK-52E cells under HG. ROS production was not suppressed by the addition of 3T3-Mt (Fig. 5c, rightmost panel). The bioactivity of 3T3-Mt is shown in Supplementary Fig. S2c.

### BM-MSC-Mt recover the expression of megalin and SGLT2 in NRK-52E cells

We next examined the functional improvement of PTECs following the addition of BM-MSC-Mt under HG. Functional improvement was assessed by the expression of megalin, as the protein transporter, and the expression of SGLT2, a member of the family of glucose transporters. The expression of megalin decreased under HG, which recovered following the addition of BM-MSC-Mt but not by the addition of 3T3-Mt (Fig. 5d). In a similar manner the expression of SGLT2 decreased under HG and recovered when BM-MSC-Mt were added (Fig. 5e).

### Isolated Mt are incorporated into PTECs of STZ rats *in vivo*

**Figure 6:**
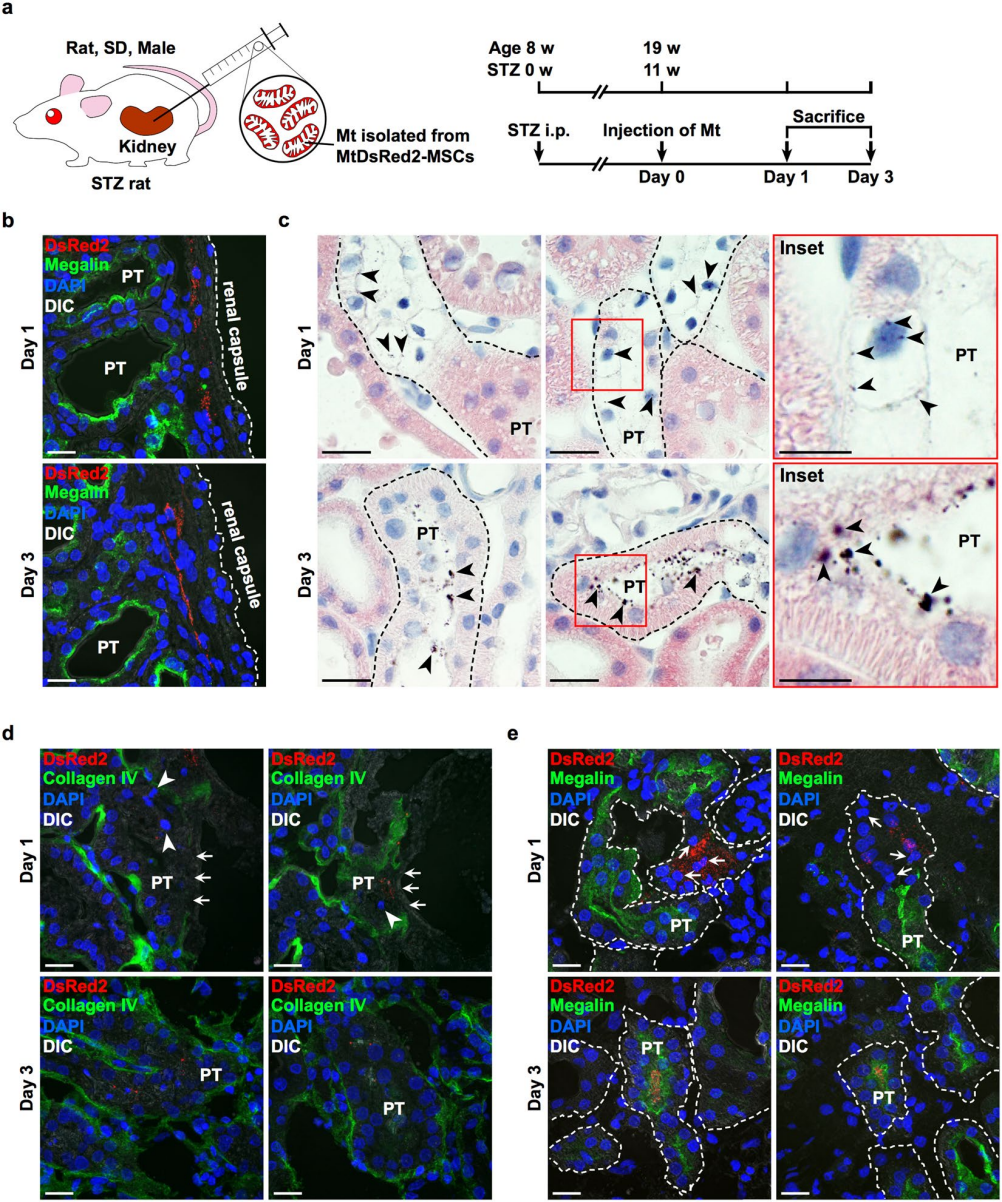
Localisation of BM-MSC-Mt injected under the renal capsule and histological improvement effects in STZ rats. (**a**) Experimental protocol for the injection of BM-MSC-Mt in STZ rats. Isolated DsRed2-Mt obtained from 1 × 10^6^ MtDsRed2-MSCs were injected under the renal capsule on the left side of the kidney. An equal volume of PBS was administered to the right kidney as the vehicle (*n* = 3 per group). (**b**) Immunofluorescence images of megalin (green) expression and localisation of isolated DsRed2-Mt (red) in the kidney of STZ rats. Nuclei are counterstained with DAPI (blue). PT, proximal tubules. Scale bar, 20 µm. (**c**) Light microscopic images of proximal tubules in STZ rats. Kidney sections were stained with H&E. Black arrowheads indicate injected isolated DsRed2-Mt stained by nickel-enhanced DAB. PT, proximal tubules. Scale bar, 20 µm in left and middle panels; 10 nm in inset. (**d**) Immunofluorescence images of collagen IV (green) expression in proximal tubules of STZ rats. Nuclei are counterstained with DAPI (blue). White arrows indicate an injured tubular basement membrane with loss or weakened expression of collagen IV. White arrowheads indicate degenerated nuclei. PT, proximal tubules. Scale bar, 20 µm. (**e**) Immunofluorescence images of megalin (green) expression in proximal tubules of STZ rats. Nuclei are counterstained with DAPI (blue). White arrows show degenerated nuclei. PT, proximal tubules. Scale bar, 20 µm.

DsRed2-Mt were injected under the renal capsule of STZ rats to clarify whether isolated Mt were incorporated into PTECs *in vivo* (Fig. 6a). The purpose of this study was to investigate Mt transfer to PTECs *in vivo* as one of the efficacy mechanisms of MSC therapy and not to clarify the therapeutic effect of BM-MSCs only by Mt transfer. Therefore, the objective of this research report was achieved with *n* = 3. A massive cluster of DsRed2-Mt was detected just under the renal capsule 1 and 3 d after the administration of DsRed2-Mt (Fig. 6b). Moreover, DsRed2-Mt were primarily detected in degenerated PTECs, in which the cytoplasmic constituents were hollowed out and nuclei had shrunk, as indicated by haematoxylin and eosin (H&E) staining 1 d after the administration (Fig. 6c–e, upper panels and Supplementary Fig. S5, upper panels). The structure of the brush border had disappeared in these PTECs, as indicated by reduced expression of megalin (Fig. 6c, upper panels and 6e, upper panels). DsRed2-Mt were also detected in the plasma membrane between adjacent injured PTECs 1 d after the administration of DsRed2-Mt. In PTECs and renal tubules where DsRed2-Mt uptake was observed, the structure of the tubular basement membrane was destroyed, as indicated by the loss or weakened expression of collagen IV (Fig. 6d, upper panels).

### Isolated Mt improve the structure of tubular basement membranes and brush borders in the PTECs of STZ rats *in vivo*

Most DsRed2-Mt were detected on the luminal side of proximal tubules 3 d after Mt administration (Fig. 6c–e, lower panels and Supplementary Fig. S5, lower panels). PTECs in these proximal tubules appeared to have normal cell structures, whereas eosinophilic cytoplasmic structures appeared and degenerated nuclei disappeared in PTECs where DsRed2-Mt were detected (Fig. 6c, lower panels and 6e, lower panels). Collagen IV expression in the tubular basement membrane and megalin expression in the brush border were observed, which is similar to intact PTECs (Fig. 6d, lower panels and 6e, lower panels). We conducted DsRed2-Mt administration under the renal capsule of normal rats to confirm whether isolated Mt were specifically incorporated into injured PTECs. DsRed2-Mt localised under the renal capsule only on the first day after administration in control rats; they were not distributed in the kidney cortex 1 and 3 d after Mt administration (Supplementary Fig. S6a). PTECs maintained the normal structure of renal tubules with megalin expression 1 and 3 d after the administration of DsRed2-Mt (Supplementary Fig. S6b).

## Discussion

In this study, we clarified that BM-MSCs transferred their Mt to diabetic-impaired PTECs *in vivo* and *in vitro*, which promoted their repair. This is the first report of Mt transfer from intravenously administered BM-MSCs to renal constituent cells *in vivo*. Incorporated Mt acted on the endogenous Mt fraction of PTECs to suppress apoptosis by regulating Mt-related factors, such as Bcl-2, Bax and PGC-1α and inhibiting ROS production, resulting in the recovery of the expression of transporters, such as megalin and SGLT2, and structural restoration of renal tubules.

PTECs reveal the highest energetic metabolism in the kidney cells which express the longevity gene Sirt 1. Down regulation of Sirt 1 suppress the metabolism of nicotinic acid, which results in impaired renal tubular epithelial cells by energy deficiency^20^. Regarding energy metabolism, Mt play an important role in cell proliferation, and a normal balance of Mt fission and fusion is required for cell cycle progression^21^. Zhan *et al*. reported that upon exposure to hyperglycaemic condition the balance of Mt fission and fusion was disrupted in human kidney cell line, which also showed Mt fragmentation induced apoptosis^13^. In addition, we investigated the impairment of endogenous Mt activity by focusing on the expression of the Mt-related proteins SOD2, Bcl-2, Bax, and PGC-1α as well as ROS production in STZ-PTECs. Cytoskeleton and the brush border were damaged in impaired PTECs, in which various transporters such as megalin and SGLT2 were downregulated by the destruction of the cell structure. Histologically, damaged PTECs were characterised by hollowed out cytoplasmic constituents with decreased eosin staining as indicated by H&E staining and shrunken nuclei *in vivo*. Impaired PTECs also showed destruction of the tubular basement membrane including the disappearance of collagen IV, and the loss of the brush border as indicated by a reduced level of megalin. Collagen IV is a major component of the tubular basement membrane^22^. In the pathological condition of DN, the renal tubular basement membrane becomes loose and thick, then splits and duplicates^23,24^. These morphological alterations were considered to induce energy deficiency due to functional disorder of endogenous Mt.

BM-MSCs exhibit a variety of functions in damaged cell repair other than Mt transfer, such as the secretion of various trophic factors or extracellular vesicles (EV) including exosomes^25–29^. Previous studies reported the therapeutic effects of intravenously administered BM-MSCs for DN treatment^19,28,30,31^. BM-MSCs inhibited inflammation and fibrosis in renal tissues and promote the regeneration of PTECs^32,33^. These effects are hypothesised to primarily arise from the paracrine effects of various trophic factors, such as vascular endothelial growth factor, hepatocyte growth factor and insulin-like growth factor^25^, as well as exosomes containing micro-RNA secreted by MSCs^25–28^. In addition, MSC-derived microvesicles (MSC-MVs) are reported to contribute to regeneration of ischemic renal tissues in the early phase of ischemia–reperfusion injury (IRI) model by providing ATP to damaged cells by transferring Mt^34^. Zou *et al*. reported that intrinsic renal scattered tubular cells (STC-like cells) repaired injured TECs by carrying and transferring Mt materials via STC-like cell-derived EV as the mitochondria cargo^35^. Oxidative stress and mitochondrial function in TECs were restored in antimycin-A-induced injured PTECs *in vitro* and unilateral renal artery stenosis model *in vivo*.

Mt transfer, a novel mechanism of action of MSCs, has recently been proposed to support the repair of endogenous Mt in target cells. Since Mt transfer from BM-MSCs to renal constituent cells had not yet been proven *in vivo*, we first examined the distribution of DsRed2-Mt derived from systemically administered MtDsRed2-MSCs in the kidney of STZ mice. DsRed2-Mt were detected in PTECs, suggesting that administered BM-MSCs transferred their own Mt into PTECs. The number of DsRed2-Mt positive PTECs per kidney was 859 cells on day3 and 776 cells on day 7 theoretically. PTECs around the PTEC which incorporated DsRed2-Mt was restored its structure by renal tubule units. These finding suggested that PTECs incorporating Mt might affect the surrounding cells and promoted their regeneration.

Next, we performed co-culture experiments with primary STZ-PTECs and BM-MSCs *in vitro* to elucidate the detailed phenomenon of Mt transfer from BM-MSCs to PTECs. DsRed2-Mt were transferred into the cytoplasm of STZ-PTECs co-cultured with BM-MSCs *in vitro*. Several studies reported that BM-MSCs transfer their own Mt to other cells through elongated cell processes referred to as tunnelling nanotubes^1,5,36–38^. Another study showed that EV containing Mt were released from astrocytes, whereby they supported cell viability and the recovery of injured neurons after stroke^39^. Morrison *et al*. reported that MSCs promoted an anti-inflammatory and highly phagocytic macrophage phenotype through EV-mediated Mt transfer in the acute respiratory distress syndrome (ARDS), which ameliorated lung injury *in vivo*^40^. Although we could not provide direct evidence for how Mt were transferred during the co-culture of these cells, BM-MSCs stretched their processes, which included DsRed2-Mt, and contacted PTECs within their vicinity. Moreover, incorporated Mt remained in impaired cells for at least 24 h, during which time it is conceivable that they acted on endogenous Mt in STZ-PTECs.

Next, we added isolated BM-MSC-derived Mt to STZ-PTECs *in vitro* to investigate Mt-specific effects while excluding the other effects of BM-MSCs, such as BM-MSC-derived exosomes as the“secretoma”. We have collected Mt by using a centrifugal force of 5800 × *g* after physically crushing BM-MSCs to differentiate from exosome effects. In general, EV that are represented by exosomes can be collected by centrifugal forces ≥100000  × *g*^41^. Therefore, we believe that negligible amounts of contamination with EV are present in our samples. Thus, the influence of contaminants can be excluded. Cell apoptosis was inhibited in STZ-PTECs after the addition of isolated Mt, which is similar to that observed when co-culturing STZ-PTECs with BM-MSCs. Kitani *et al*. showed that Mt isolated from human uterine endometrial gland-derived mesenchymal cells improved the oxygen consumption rate, ATP production and spare respiratory capacity in endometrial gland-derived mesenchymal cells in which endogenous Mt were depleted^42^. These findings suggest that in the present study, incorporated Mt derived from BM-MSCs might complement the function of endogenous Mt in STZ-PTECs to suppress apoptosis of damaged PTECs.

We investigated the action of isolated Mt on endogenous Mt activity by focusing on the expression of the Mt-related proteins SOD2, Bcl-2, Bax and PGC-1α, as well as ROS production in STZ-PTECs. SOD is a major ROS-scavenging enzyme that catalyses the immediate conversion from superoxide anion to hydrogen peroxide^43^. SOD2 is particularly important for maintaining homeostasis and the survival of cells^9^. Mice with a partial SOD2 deficiency exhibit oxidative stress and renal interstitial inflammation, which accelerates renal senescence and salt-sensitive hypertension^44^. Bcl-2, which localises in the outer membrane of Mt^45^, suppresses the activation of pro-apoptotic factors such as Bax and Bak, which release cytochrome *c* from Mt into the cytoplasm and enhance apoptotic reactions. Bcl-2 overexpression also enhances SOD and catalase enzymatic activities in neural cells, indicating that it regulates antioxidant pathways^46–48^. Intranuclear translocation of PGC-1α has been reported to promote the biosynthesis of Mt^49,50^. PGC-1α expression increased and nuclear translocation occurred in serum-free environment^51^ and under HG condition^50^. These reactions are thought to be compensatory actions for impaired Mt. In this study, Bcl-2 expression was decreased and Bax expression was increased, and hyperexpression and nuclear transition of PGC-1α occurred *in vitro*. These changes were corrected by the addition of BM-MSC-Mt (Fig. 5a), which indicated that isolated Mt supplied energy to PTECs and released the biosynthesis of Mt. ROS are generated by Mt in most mammalian cells, and excessive ROS production induces oxidative damage in mitochondrial proteins, membranes and DNA, which impairs the ability of Mt to synthesise ATP^43^. Park *et al*. reported that administration of SOD suppressed DN progression in OLETF rats, a model of type 2 diabetes, by scavenging ROS in Mt and altering signal transduction^52^. We observed that Mt were incorporated rapidly into PTECs (within 24 h) and affected SOD2, Bcl-2 and Bax expression in STZ-PTECs, thereby suppressing ROS production. We postulate that the Mt-enriched fraction of STZ-PTECs-Mt (+) contains both endogenous Mt and Mt derived from BM-MSCs. Thus, Mt derived from BM-MSCs, which included higher amounts of SOD2 and Bcl-2, might complement the function of impaired endogenous Mt in STZ-PTECs.

Mt isolated from BM-MSCs were incorporated into injured renal tubules *in vivo* 1 d after the administration of Mt, whereby they induced histological improvements of proximal tubules in STZ rats within 3 d. According to previous reports, most of the intravenously administered Mt were trapped in the lungs and did not distribute to the kidneys^53^. From this result, we considered that it is difficult to assess the effect of MSC-derived isolated Mt on kidneys by systematic intravenous administration. If isolated Mt were administered directly to the renal artery, these Mt could diffuse into renal tissues due to physiological blood flow. However, the blood flow velocity is very fast in the renal artery, so this may interfere with effective diffusion of administered Mt. Furthermore, there are high barriers of endothelial cells of the glomerular capillary and peritubular epithelial capillary to acts on PTECs. These barriers were expected to reduce the number and activity of administered Mt. Since the objected was to confirm the cytoprotective effect of MSC-derived isolated Mt on PTECs, we administered Mt under the renal capsule as a more reliable method and evaluated their effect on the kidney. Moreover, we have previously evaluated the effectiveness of intravenous administration of rat BM-MSCs in STZ mice^28^. The purpose of this study was to elucidate the phenomenon of Mt transfer as an efficacy mechanism of BM-MSCs. Since it is technically difficult to administer isolated Mt under the renal capsule of mice, leading to a lack of reproducibility, we used a rat model instead of mice for the under renal capsule administration experiment of isolated Mt. Megalin is the scavenger receptor which is highly expressed on the luminal side of PTECs and reabsorbs and metabolises various ligand proteins that filter the glomeruli. Megalin ransports β2 - microglobulin and AGE into cells and metabolising them. SGLT2 is a transporter that is distributed in brush border membranes of PTECs and incorporates glucose into cells by active transport of SGLT2. In this study, Mt transfer caused structural restoration of PTECs, which resulted in recovering the expression of megalin and SGLT2 as the structural marker in PTECs. However, the effect of Mt transfer on their functionality of the absorption was unknown.

Isolated Mt were incorporated into STZ-PTECs more than Control-PTECs *in vitro*. Islam *et al*. reported that BM-MSCs transferred their Mt to alveolar epithelial cells more than controls in a lipopolysaccharide-induced lung injury model^1^, while Ahmad *et al*. found that BM-MSCs transferred their Mt to injured bronchial epithelial cells more than undamaged cells when BM-MSC therapy was used to treat a rotenone-induced (Mt stress) bronchial injury mouse model^5^. Thus, our results suggest that isolated Mt were incorporated into PTECs in response to endogenous mitochondrial damage of PTECs. Additionally, isolated Mt were incorporated into STZ-PTECs in a dose-dependent manner, which resembles the incorporation of isolated Mt from BM-MSCs into the human breast adenocarcinoma cell line MDA-MB-231^54^. This previous study also reported a decrease in ATP production and cell proliferation when higher amounts of isolated Mt were added to cells, suggesting that Mt are not only a supplier of energy such as ATP, but also a major source of ROS which may cause cytotoxicity by inducing oxidative stress. Therefore, the incorporation of an appropriate amount of Mt is necessary to recover cell functions without damaging cells.

In conclusion, we demonstrated that BM-MSCs ameliorate impaired PTECs by Mt transfer in a DN model. BM-MSC-derived isolated Mt were transferred to damaged PTECs *in vivo* and *in vitro*. Incorporated Mt suppressed apoptosis of PTECs *in vitro*, either by direct co-culturing with BM-MSCs or following the addition of isolated Mt. Mt isolated from BM-MSCs rescued endogenous Mt function by regulating SOD2, Bcl-2, Bax and PGC-1α expression and suppressing ROS production, which inhibited apoptosis of PTECs *in vitro*. Isolated Mt passed through the structurally damaged tubular basement membrane and were incorporated into the cytoplasm, plasma membrane and gap between adjacent injured PTECs that had lost their structural brush border. Incorporated Mt may repair PTECs *in vivo*, which could be accompanied by the restoration of renal tubule structures. According to these findings, Mt transfer plays an important role in the therapeutic effects of BM-MSCs toward DN treatment.

## Methods

### Animal models of diabetes

Eight-week-old male C57BL/6 mice and 8-week-old male Sprague-Dawley (SD) rats were purchased from Sankyo Lab Service (Tokyo, Japan). Diabetic mice were obtained by a single intraperitoneal injection of 150 mg/kg STZ (Wako Pure Chemical Industries, Osaka, Japan) dissolved in citrate buffer (pH 4.5) into 8-week-old C57BL/6 mice. Diabetic rats were obtained by a single tail vein injection of 55 mg/kg of STZ into 8-week-old SD rats. Control mice and rats were treated with citrate buffer via intraperitoneal and intravenous injections, respectively. Successful establishment of the diabetes model was confirmed one week after STZ injection by measuring blood glucose levels of ≥400 mg/dl for STZ-diabetic mice, or ≥300 mg/dl for STZ-diabetic rats. All mice and rats were housed at a constant temperature and humidity with a 12-h light/dark cycle and given free access to food and water. All methods for animal experiments were performed in accordance with the relevant guidelines and regulations of the animal experiment committee of Sapporo Medical University (Sapporo, Japan). All experimental protocols and studies were approved by the animal experiment committee of Sapporo Medical University.

### Isolation and Characterization of BM-MSCs

Bone marrow cells were harvested from 8-week-old SD rats and cultured as described previously^55^. Immunophenotyping and the differentiation potential of BM-MSCs were then determined.

### Fluorescence labelling of Mt in BM-MSCs

To specifically label endogenous Mt in BM-MSCs, the DsRed2 protein (which is expressed specifically in Mt) was transfected into BM-MSCs using a lentiviral system (Invitrogen, Carlsbad, CA), as described previously^56^. Briefly, a total of 2 × 10^3^ BM-MSCs were seeded into 3.5-cm culture dishes as passage 1. After 24 h, 1 mL of complete medium containing lentiviruses was added to BM-MSCs at a multiplicity of infection of 200. After 24 h, the medium was replaced with fresh complete medium. BM-MSCs were cultured until passage 3 (MtDsRed2-MSCs). The expression of DsRed2 in MtDsRed2-MSCs was observed with Axio Observer Z1 (Carl Zeiss, Jena, Germany). The transfection efficiency of DsRed2 was assessed by using Aria III flow cytometer (Becton Dickinson, Franklin Lakes, NJ).

### Intravenous administration of MtDsRed2-MSCs to STZ mice

Eight weeks after STZ injection, mice were administered twice with 1 × 10^4^ MtDsRed2-MSCs (STZ-MtDsRed2-MSCs) per gram of body weight via the tail vein every two weeks. Renal tissues were obtained at the optimised time after the administration of MtDsRed2-MSCs.

### Isolation and characterization of Mt derived from BM-MSCs

Mt were isolated from BM-MSCs, MtDsRed2-MSCs and NIH-3T3 cells (ATCC, Manassas, VA) by centrifugation methods, as described previously^42^. Cells were collected from culture dishes with trypsin (Thermo Fisher Scientific, Waltham, MA) and pelleted by centrifugation at 2300 × *g* for 5 min. The cell pellet was resuspended in homogenisation buffer containing 20 mM HEPES-KOH (pH 7.4), 220 mM mannitol, 70 mM sucrose and a protease inhibitor cocktail (Sigma-Aldrich, St. Louis, MO) at a density of 1.0 × 10^7^ cells/mL and incubated on ice for 5 min. Next, cells were crushed by 60 strokes using a 27-gauge needle on ice. Crushed cells were centrifuged three times at 400 × *g* for 5 min to remove unbroken cells. Mt were pelleted by centrifugation at 5800 × *g* for 5 min. The expression of DsRed2 in isolated Mt was confirmed by confocal laser-scanning microscopy (Nikon A1; Nikon, Tokyo, Japan). Morphological findings of isolated Mt were observed by transmission electron microscopy (H7650; Hitachi High-Technologies Corporation, Tokyo, Japan). Viability of isolated Mt was measured using the Extracellular O_2_ Consumption Assay (Abcam, Cambridge, UK) following the manufacturer’s instructions.

### Primary culture of rat PTECs

PTECs were prepared according to a reported protocol^28^. Primary cultured PTECs were obtained from Control or STZ rats. The characterization of PTECs was evaluated by the expression of LTL, which is a PTECs marker^57^.

### Culture conditions of NRK-52E cells

NRK-52E cells were purchased from the JCRB Cell Bank (Osaka, Japan), and were cultured at a density of 9 × 10^3^ cells/cm^2^ with low glucose DMEM (5.6 mM D-glucose) containing 10% foetal bovine serum (FBS; Sigma-Aldrich) and 1% penicillin–streptomycin (PS; Thermo Fisher Scientific) at 37 °C in 5% CO_2_. High glucose DMEM was prepared by supplementing D-glucose (KANTO CHEMICAL, Tokyo, Japan) to a final concentration of 55 mM. NRK-52E cells were cultured in low or high glucose medium for 60 h followed by starvation with serum-free low glucose medium for 24 h.

### Co-culture of PTECs and BM-MSCs or MtDsRed2-MSCs

Forty-eight h after the initial medium change, PTECs were co-cultured with BM-MSCs or MtDsRed2-MSCs at a density of 3.5 × 10^3^ cells/cm^2^ for 96 h. Ninety-six h after co-culture with BM-MSCs or MtDsRed2-MSCs, cells were fixed with 4% paraformaldehyde (PFA).

### Addition of isolated Mt to PTECs and NRK-52E cells

Forty-eight h after the initial medium change, various amounts of BM-MSC-Mt or MtDsRed2-MSC-Mt were added to PTECs for 96 h at 37 °C in 5% CO_2._ Cells were then fixed with 4% PFA. Sixty h after high glucose stimulation of NRK-52E cells, BM-MSC-Mt or NIH-3T3-Mt were added to NRK-52E cells and the cells were cultured for a further 24 h at 37 °C in 5% CO_2._

### Time-lapse imaging

Twelve h after co-culturing PTECs with MtDsRed2-MSCs or the addition of DsRed2-Mt, time-lapse observations and imaging of phase contrast and fluorescence were commenced with Axio Observer Z1 (Carl Zeiss) at 37 °C in 5% CO_2_ using a stage top incubator (Tokai Hit, Fujinomiya, Japan).

### Immunofluorescence staining

Kidney tissues, which were immersed in 4% PFA and cryosectioned, STZ-PTECs and NRK-52E cells were incubated with primary and secondary antibodies (Supplementary Table S1). Nuclei were stained with DAPI and observed by confocal laser-scanning microscopy (Nikon A1). The fluorescence signal intensity in NRK-52E cells was measured by confocal laser-scanning microscopy (Nikon A1).

### TUNEL staining

Fixed cells were permeabilised with PBS containing 0.3% Triton X-100 (PBST). Next, TUNEL staining was performed to evaluate the apoptotic state of PTECs using the DeadEnd Fluorometric TUNEL System (Promega, Madison, WI). Nuclei were stained with DAPI (Dojindo Laboratories, Kumamoto, Japan). Cells were observed by confocal laser-scanning microscopy (Nikon A1).

### Immunoblotting

Protein expression of STZ-PTECs and NRK-52E cells was analysed by immunoblotting, as described in detail in the Supplementary Methods (Supplementary Table S2).

### Measurement of ROS levels

ROS levels in STZ-PTECs or NRK-52E cells were measured using CellROX Oxidative Stress Reagents (Thermo Fisher Scientific). Cells were cultured in 96-well black plate with a clear bottom (Greiner Bio-One, Kremsmünster, Austria). ROS levels were measured 96 h after the addition of isolated Mt in STZ-PTECs and 24 h after the addition of isolated Mt in NRK-52E cells. Each sample was measured in triplicate. Cells were treated with CellROX Oxidative Stress Reagents for 30 min, and then the absorbance at 520 nm was measured using a microplate reader (Infinite M1000 Pro; TECAN, Männedorf, Switzerland). Fluorescence images and fluorescence signal intensities were obtained by confocal laser-scanning microscopy (Nikon A1).

### Administration of isolated Mt under the renal capsule of STZ rats

Eleven weeks after STZ injection, DsRed2-Mt obtained from 1 × 10^6^ MtDsRed2-MSCs were suspended in 100 µl of sterile phosphate-buffered saline (PBS) and injected under the renal capsule of the left kidney of STZ rats using a 200 µL micropipette tip. One × 10^4^ cells/g body weight of BM-MSCs were intravenously administered in mice. Since the body weight of rats was around 200 g at the point of administration, 2 × 10^6^ cells of BM-MSCs per individual would be administered when converting this rate into rats. That is, the number of BM-MSCs effecting one kidney is assumed to be 1 × 10^6^ cells. Therefore, we isolated Mt from this number of BM-MSCs and administered under the renal capsule. An equal volume of PBS was administered to the right kidney as the vehicle. Renal tissues were obtained 1 day and 3 d after Mt injection.

### Nickel-enhanced diaminobenzidine (DAB) staining

Paraffin-embedded kidney tissues were cut into 5-µm sections and deparaffinised. Sections were permeabilised with PBST for 3 days at 4 °C and incubated with primary antibodies against DsRed2 overnight at 4 °C. Next, sections were incubated with the Envision + System horseradish peroxidase-labelled polymer anti-rabbit IgG (K4002, Dako, Glostrup, Denmark) for 30 min at room temperature (RT), then visualised with 0.01% DAB containing 1% nickel in 0.05 M Tris-HCl buffer (pH 7.6) for 60 min at RT. Sections were also stained with H&E and observed under light microscopy (NIS element BR 3.0; Nikon).

### Statistical analysis

Data are represented as the mean ± s.e.m. Statistical analysis was carried out using an unpaired t-test or one-way analysis of variance (ANOVA) followed by Bonferroni’s test for post-hoc comparisons between groups. Statistical analysis was performed using R Statistical Software (Foundation for Statistical Computing, Vienna, Austria) and differences were considered significant at *P* < 0.05.

## Supplementary information

Supplementary information

Supplementary Video S1

Supplementary Video S2

Supplementary Video S3

Supplementary Video S4

## Data Availability

All data generated or analysed during this study are included in this published article and its Supplementary Information Files.
