# Supplementary material for: Mitochondria transfer from mesenchymal stem cells structurally and functionally repairs renal proximal tubular epithelial cells in diabetic nephropathy in vivo
Source: Sci Rep. 2019 Mar 26;9:5184. doi: 10.1038/s41598-019-40163-y (PMC6435708; doi:10.1038/s41598-019-40163-y)

Mitochondria transfer from mesenchymal stem cells structurally and functionally repair renal proximal tubular epithelial cells in diabetic nephropathy *in vivo*

Naoto Konari, Kanna Nagaishi*, Shin Kikuchi & Mineko Fujimiya

*Corresponding author

SUPPLEMENTARY INFORMATION

1. Supplementary Results

2. Supplementary Methods

3. Supplementary References

4. Supplementary Figure, Table and Video Legends

5. Supplementary Tables

6. Supplementary Figures

7. Supplementary Videos

Supplementary Results

Characterization of BM-MSCs. BM-MSCs expressed cluster of differentiation (CD) 90 and CD44 cell surface antigens, whereas CD45, CD34, CD11b, CD105 and human leukocyte antigen-DR (HLA-DR) were not expressed (Supplementary Fig. S1a, upper panels). MSCs exhibited osteogenic, adipose and chondrogenic differentiation abilities (Supplementary Fig. S1b).

Characterization of MtDsRed2-MSCs. Endogenous Mt in BM-MSCs were labelled with the DsRed2 fluorescence protein by gene transfection (MtDsRed2-MSCs). The DsRed2-expressed gene was successfully transfected into BM-MSCs at a rate of ~80%. Almost all of endogenous Mt expressed DsRed2 (DsRed2-Mt) (Supplementary Figs. S1c and S1d). MtDsRed2-MSCs expressed CD90 and CD44 cell surface antigens, whereas CD45, CD34, CD11b, CD105 and HLA-DR were not expressed, and these observations were similar to that observed for non-transfected-BM-MSCs (Supplementary Fig. S1a, lower panels). Cell proliferation was similar in MtDsRed2-MSCs and non-transfected-BM-MSCs (Supplementary Fig. S1e).

Supplementary Methods

Isolation and culturing of rat BM-MSCs. Bone marrow was collected from 8-week-old male SD

rats. BM-MSCs were harvested by adherent cultures of bone marrow cells, as described previously[1]. Bone marrow cells were harvested from femurs, tibias and humeri by flushing whole bone marrow with complete α-Modified Eagle's Medium (α-MEM; Invitrogen) containing 15% foetal bovine serum (FBS; Sigma-Aldrich) and 1% penicillin–streptomycin (PS; Thermo Fisher Scientific). Single-cell suspensions were filtered through a 100-μm sterile filter (Becton Dickinson) and plated in 150-cm^2 dishes. Cells were grown in complete α-MEM containing 15% FBS and 1% PS at 37 °C and 5% CO_2. Three d after bone marrow cells seeding, the medium was replaced with fresh medium, and adherent cells grown to 80% confluency were defined as passage 0. Cells in passage 3 were used for experiments.

Characterisation of rat BM-MSCs and MtDsRed2-MSCs. The immune phenotype of BM-MSCs and MtDsRed2-MSCs was determined using Aria III flow cytometer (Becton Dickinson) and rat surface antigen-specific antibodies CD90, CD44, CD45, CD34, CD11b, CD105 and HLA-DR (Supplementary Table S3).

Differentiation ability of BM-MSCs. The differentiation ability of rat BM-MSCs was confirmed using the AdipoInducer Reagent (for animal cell) (Takara Bio Inc., Shiga, Japan) for adipogenic

differentiation ability and the Rat Mesenchymal Stem Cell Functional Identification Kit (R&D systems Inc., Minneapolis, MN) for osteogenic and chondrogenic differentiation abilities, following the manufacturer's instructions. Adipocytes, osteocytes and chondrocytes were detected by immunofluorescence staining using the goat anti-FABP4 antibody, mouse anti-osteocalcin antibody and goat anti-aggrecan antibody, respectively. Alexa 488 conjugated anti-goat IgG or Alexa 488 conjugated anti-mouse IgG were used as secondary antibodies. Nuclei were stained with DAPI and observed by confocal laser-scanning microscopy (Nikon A1).

Proliferation assay of BM-MSCs and MtDsRed2-MSCs. Proliferation of BM-MSCs and MtDsRed2-MSCs was analysed using the Cell Counting Kit-8 (CCK-8; Dojindo Laboratories). A total of 3×10^3 cells were cultured in each well of a 96-well cell culture plate (Corning Costar; Sigma-Aldrich) for 48 and 72 h. Triplicate wells were used for each sample. Cells were treated with 10 μL of tetrazolium salt (WST-8) in each well for another 2 h. The absorbance at 450 nm (A450) was measured with a microplate reader (Infinite M1000 Pro; TECAN).

Isolation and primary culturing of rat PTECs. PTECs were prepared according to a protocol reported previously[2] for evaluating Mt transfer from BM-MSCs to PTECs or the direct effect of

isolated Mt alone on PTECs *in vitro*. Kidneys were removed from Control or STZ rats and minced with scissors in a solution of 1 mg/mL collagenase (Sigma-Aldrich) diluted in sterile PBS and incubated for 30 min at 37 °C. Dissolved tissue was filtered with a 100-μm pore cell strainer and centrifuged at 400 × *g* for 5 min. The cell pellet was treated with RBC lysis buffer (Qiagen, Hilden, Germany). Next, cells were plated onto appropriately sized culture dishes or chamber slides at a density of 2.5×10^5 cells/cm^2 and cultured with DMEM/F-12 (Thermo Fisher Scientific) containing 10% FBS, 1% PS, Insulin-Transferrin-Selenium-X (Invitrogen), prostaglandin E1 (7.1×10^{-8} M; Sigma-Aldrich), triiodothyronine (2.0×10^{-9} M; Sigma-Aldrich) and dexamethasone (5.09×10^{-8} M; Sigma-Aldrich), and incubated at 37 °C in 5% CO_2 for 48 h. The medium was replaced 48 h after initial plating. Phase contrast images of cultured PTECs were observed with an Eclipse TE200 (Nikon).

Transmission electron microscopy. The pellet of isolated Mt from BM-MSCs was fixed in 2% paraformaldehyde (PFA) and 2% glutaraldehyde (Wako Pure Chemical Industries) in 0.1 M cacodylate buffer overnight at 4 °C. Next, the pellet was post-fixed with 1% osmium tetroxide (TAAB Laboratories Equipment, Aldermaston, UK) and embedded with epoxy resin (EPON812, TAAB Laboratories Equipment). Ultra-thin sections (70 nm) were cut with an ultramicrotome (RMT,

Tucson, AZ), stained with uranyl acetate and lead citrate and observed by transmission electron microscopy (Hitachi High-Technologies Corporation).

Immunofluorescence staining. Kidney samples were fixed with 4% PFA, immersed in O.C.T. compound (Sakura Finetek USA, Torrance, CA) and cryosectioned to 20 μm. Frozen sections were permeabilised with PBST for 3 d at 4 °C. After permeabilisation, sections were incubated in 2.5% donkey serum in PBS for 1 h at RT and then incubated with primary antibodies (Supplementary Table S1) overnight at 4 °C. Sections were then washed with PBST and incubated with secondary antibodies (Supplementary Table S1) for 2 h at RT. Nuclei were stained with DAPI (Dojindo Laboratories). Sections were observed by confocal laser-scanning microscopy (Nikon A1).

PTECs and NRK-52E cells cultured *in vitro* were fixed with 4% PFA and permeabilised with 0.3% PBST for 10 min at RT. Cells were washed with PBS and incubated in Biotin-Blocking Solution (ScyTek Laboratories, Logan, UT) to block endogenous biotin expression. Cells were then washed with PBS and incubated with primary antibodies (Supplementary Table S1) for 1 h at RT, and subsequently washed again with PBS and incubated with secondary antibodies (Supplementary Table S1) for 30 min at RT. Nuclei were stained with DAPI. Cells were observed by confocal laser-scanning microscopy (Nikon A1).

Immunoblotting. Protein expression in STZ-PTECs and NRK-52E cells was analysed by immunoblotting. Protein expression in cytosol-enriched and Mt-enriched fractions of STZ-PTECs were analysed separately by obtaining these fractions using centrifugation methods. Cells were collected and pelleted by centrifugation at 2300 × *g* for 5 min. The Mt fraction was collected as described previously[3]. The supernatant of the cell pellet was centrifuged at 10000 × *g* for 15 min to obtain the cytosol-enriched fraction. Each fraction was lysed in the radioimmunoprecipitation assay buffer containing 20 mM Tris-HCl, pH 7.4, 150 mM sodium chloride, 1 mM EDTA (Sigma-Aldrich), pH 8.0, 0.1% (w/v) sodium dodecyl sulfate, 0.1% sodium deoxycholate, 1% Triton X-100, complete Mini™ (Roche Diagnostics, Basel, Switzerland) and Phos STOP (Roche Diagnostics). After measuring protein concentrations using the bicinchoninic acid Protein Assay Kit (Thermo Fisher Scientific), 5–40 μg of protein obtained from each fraction was separated on a 12% denaturing polyacrylamide gel before transfer to a polyvinylidene difluoride membrane. After blocking with 5% non-fat dry milk in Tris-buffered saline supplemented with Tween 20, the membrane was incubated with primary antibodies (Supplementary Table S2) overnight at 4 °C. Subsequently, the membrane was incubated with horseradish peroxidase-conjugated secondary antibodies (Supplementary Table S2) for 1 h at RT. Immunoreactivity was developed using an Enhanced Chemiluminescence Kit (GE

Healthcare, Amersham, UK).

Supplementary References

1 Javazon, E. H., Colter, D. C., Schwarz, E. J. & Prockop, D. J. Rat marrow stromal cells are more sensitive to plating density and expand more rapidly from single-cell-derived colonies than human marrow stromal cells. *Stem Cells* **19**, 219-225, doi:10.1634/stemcells.19-3-219 (2001).

2 Nagaishi, K. *et al.* Mesenchymal stem cell therapy ameliorates diabetic nephropathy via the paracrine effect of renal trophic factors including exosomes. *Sci Rep* **6**, 34842, doi:10.1038/srep34842 (2016).

3 Kitani, T., Kami, D., Matoba, S. & Gojo, S. Internalization of isolated functional mitochondria: involvement of macropinocytosis. *Journal of cellular and molecular medicine* **18**, 1694-1703, doi:10.1111/jcmm.12316 (2014).

Supplementary Figure Legends

Supplementary Figure S1. Characteristics of BM-MSCs and MtDsRed2-MSCs. (**a**) Flow cytometry analysis of cell surface marker expression in non-transfected-BM-MSCs and MtDsRed2-MSCs. The red histogram summarizes the results for the population stained with the target antibody, whereas the

blue histogram summarizes the results for the population stained with the isotype control antibody. Experiments were repeated three times independently. (**b**) Adipogenic, osteogenic and chondrogenic differentiation of BM-MSCs. Nuclei are counterstained with DAPI (blue). Scale bar, 20 μm. (**c**) Flow cytometry analysis of DsRed2 expression in MtDsRed2-MSCs. The red histogram summarizes the results for MtDsRed2-MSCs and the blue histogram summarizes the results for non-transfected-BM-MSCs. (**d**) Morphological findings of BM-MSCs, as indicated by phase contrast images and expression of DsRed2 in BM-MSCs. Non-transfected-BM-MSCs are shown in the left panel and MtDsRed2-MSCs are shown in the middle panel. Scale bar, 100 μm. A single cell image of MtDsRed2-MSCs is shown in the right panel under high magnification. A nucleus is counterstained with DAPI (blue). Scale bar, 10 nm. (**e**) Cell proliferative ability of non-transfected-BM-MSCs and MtDsRed2-MSCs. Absorbance at 450 nm was measured 0, 48 and 72 h after commencing cell culturing. Data are shown as mean ± s.e.m. (n = 3 per group).

Supplementary Figure S2. Images of isolated DsRed2-Mt and viability of isolated Mt (**a**) Fluorescence image of DsRed2-Mt isolated from MtDsRed2-MSCs. Scale bar, 5 μm. (**b**) A transmission electron microscopic image of DsRed2-Mt isolated from MtDsRed2-MSCs. Scale bar, 200 nm. (**c**) Oxygen consumption rate of isolated Mt. Rates of the slope signal change were

normalized against the initial intensity.

Supplementary Figure S3. Quantity-dependent incorporation rate of DsRed2-Mt in Control-PTECs and STZ-PTECs. Differences in incorporation rates of DsRed2-Mt into Control-PTECs and STZ-PTECs are shown for each additional amount of DsRed2-Mt. The amount of Mt shows the ratio to the number of Control-PTECs or STZ-PTECs when isolated Mt were added. Data are shown as mean ± s.e.m. (n = 3 per group). *P < 0.05 STZ-PTECs vs. Control-PTECs using the unpaired t-test.

Supplementary Figure S4. SOD1 expression in the cytosol-enriched fraction of STZ-PTECs cultured with or without isolated Mt *in vitro.* (**a**) Western blotting analysis of SOD1 expression in STZ-PTECs 24 h after the addition of isolated Mt (n = 4 per group). Cropped images of immunoblots are shown here, and full-length blots are shown in Supplementary Fig. S7c. (**b**) Densitometric analysis of SOD1 expression in STZ-PTECs 24 h after the addition of isolated Mt. Data are shown as mean ± s.e.m. (n = 4 per group).

Supplementary Figure S5. Three-dimensional images of kidney sections of STZ rats injected with isolated DsRed2-Mt under the renal capsule. Immunofluorescence images of megalin expression

(green) and DsRed2-Mt (red) in proximal tubules. Nuclei are counterstained with DAPI (blue). Red and yellow lines indicate the same position in images at each time point.

Supplementary Figure S6. Distribution of isolated Mt in the kidney of control rats injected with DsRed2-Mt under the renal capsule. (**a**) Experimental protocol for the injection of isolated Mt in control rats. Isolated DsRed2-Mt obtained from 1×10^6 MtDsRed2-MSCs were injected under the renal capsule in each side of the kidney (n = 3 per group). (**b**) Immunofluorescence images of megalin (green) expression and DsRed2-Mt (red) in proximal tubules. Nuclei are counterstained with DAPI (blue). PT, proximal tubules. Scale bar, 20 µm.

Supplementary Figure S7. Full unedited gels from Figs. 4a, 5a and S4a. (**a**) Full unedited gel from Fig. 4a. (**b**) Full unedited gel from Fig. 5a. (**c**) Full unedited gel from Supplementary Fig. S4a.

Supplementary Table Captions

Supplementary Table S1. Primary and secondary antibodies used for immunofluorescence staining.

Supplementary Table S2. Primary and secondary antibodies used for western blotting analysis.

Supplementary Table S3. Primary and secondary antibodies used for flow cytometry analysis.

Supplementary Video Legends

Supplementary Video S1. Time-lapse observation of STZ-PTECs co-cultured with MtDsRed2-MSCs. The time-lapse observation started 12 h after commencing the co-culture. The yellow arrow tracks the same MtDsRed2-MSC-derived Mt (red).

Supplementary Video S2. Time-lapse observation of STZ-PTECs co-cultured with MtDsRed2-MSCs. The time-lapse observation started 12 h after commencing the co-culture. The yellow arrow tracks the same MtDsRed2-MSC-derived Mt (red).

Supplementary Video S3. Time-lapse observation of STZ-PTECs co-cultured with DsRed2-Mt (red) isolated from MtDsRed2-MSCs. The time-lapse observation started 12 h after the addition of DsRed2-Mt. The yellow arrow tracks the same isolated DsRed2-Mt (red).

Supplementary Video S4. Time-lapse observation of STZ-PTECs co-cultured with DsRed2-Mt (red) isolated from MtDsRed2-MSCs. The time-lapse observation started 12 h after the addition of DsRed2-Mt.

Supplementary Table S1. Primary and secondary antibodies used for immunofluorescence staining.

Antibody	Species	Reactivity	Manufacturer	No.	Dilution
Primary antibodies					
DsRed2	Rab		Clontech Laboratories	632496	1:250
Collagen IV	Rab	Ms, Rt	Abcam	ab19808	1:100
Megalin	Rab	Ms, Hu, Mk	Abcam	ab76969	1:500 (T)<br>1:200 (C)
Megalin	Goat	Ms, Rt	Santa Cruz Biotechnology	Sc-16478	1:50
Biotinylated LTL			Vector Laboratories	B-1325	1:30
SGLT2 (SLC5A2)	Rab	Hu	EPIGENTEK	A63450	1:100
PGC-1α	Rab	Ms, Rt, Hu	Novus Biologicals	NBP1-04676	1:200
Secondary antibodies					
Alexa 488 conjugated anti-rabbit IgG	Dky	Rab	Jackson ImmunoResearch Laboratories	711-545-152	1:500 (T)<br>1:200 (C)
Cy3 conjugated Anti-goat IgG	Dky	Gt	MERCK	AP180C	1:500
Alexa 488 Conjugated streptavidin			Jackson ImmunoResearch Laboratories	016-540-084	1:500

Ms, mouse; Rt, rat; Rab, rabbit; Hu, human; Mk, monkey; Gt, goat; Dky, donkey; (T), for tissues; (C), for cells

Supplementary Table S2. Primary and secondary antibodies used for western blotting analysis.

Antibody	Species	Molecular weight (kDa)	Manufacturer	No.	Dilution
Primary antibodies					
SOD1	Rab	17	Abcam	ab16831	1:2000
SOD2	Rab	22	Cell Signaling Technology	13141	1:2000
Bcl-2	Ms	26	Santa Cruz Biotechnology	sc-7382	1:200
Bax	Ms	21	Proteintech	60267-1-lg	1:2000
β-actin	Ms	42	Sigma-Aldrich	A5441	1:5000
VDAC1	Rab	31	Abcam	ab154856	1:5000
Secondary antibodies					
HRP conjugated anti-mouse IgG	Sh		GE healthcare	NA931V	1:2000
HRP conjugated anti-rabbit IgG	Dky		GE healthcare	NA934V	1:2000

Ms, mouse; Rab, rabbit; Sh, sheep; Dky, donkey.

Supplementary Table S3. Primary and secondary antibodies used for flow cytometry analysis.

Antibody	Species	Reactivity	Manufacturer	No.	Dilution
Primary antibodies					
CD90	Ms	Rt, Ms	BioLegend	202501	1:100
CD44	Ms	Rt	BioLegend	203901	1:100
CD45	Ms	Rt	BioLegend	202201	1:100
CD34	Rab	Hu, Rt, Ms	Bioss	bs-2038R	1:100
CD11b	Ms	Rt	Chemicon	CBL1512	1:100
CD105	Ms	Hu, Rt, Ms, Dg, Mk	Abcam	ab156756	1:100
HLA-DR	Ms	Rt	BioLegend	205301	1:100
Isotype control					
Mouse IgG1, κ	Ms		BD Bioscience	556648	1:100
Mouse IgG2a, κ	Ms		BD Bioscience	556651	1:100
Mouse IgG2b, κ	Ms		BioLegend	401201	1:100
Rabbit IgG	Rab		BioLegend	910801	1:100
Secondary antibodies					
FITC conjugated anti-mouse IgG	Gt	Ms	MERCK	AP127F	1:50
FITC conjugated anti-rabbit IgG	Dky	Rab	MERCK	AP182F	1:50

Ms, mouse; Rt, rat; Rab, rabbit; Hu, human; Dg, dog; Mk, monkey; Gt, goat; Dky, donkey.

Supplementary Figure S1

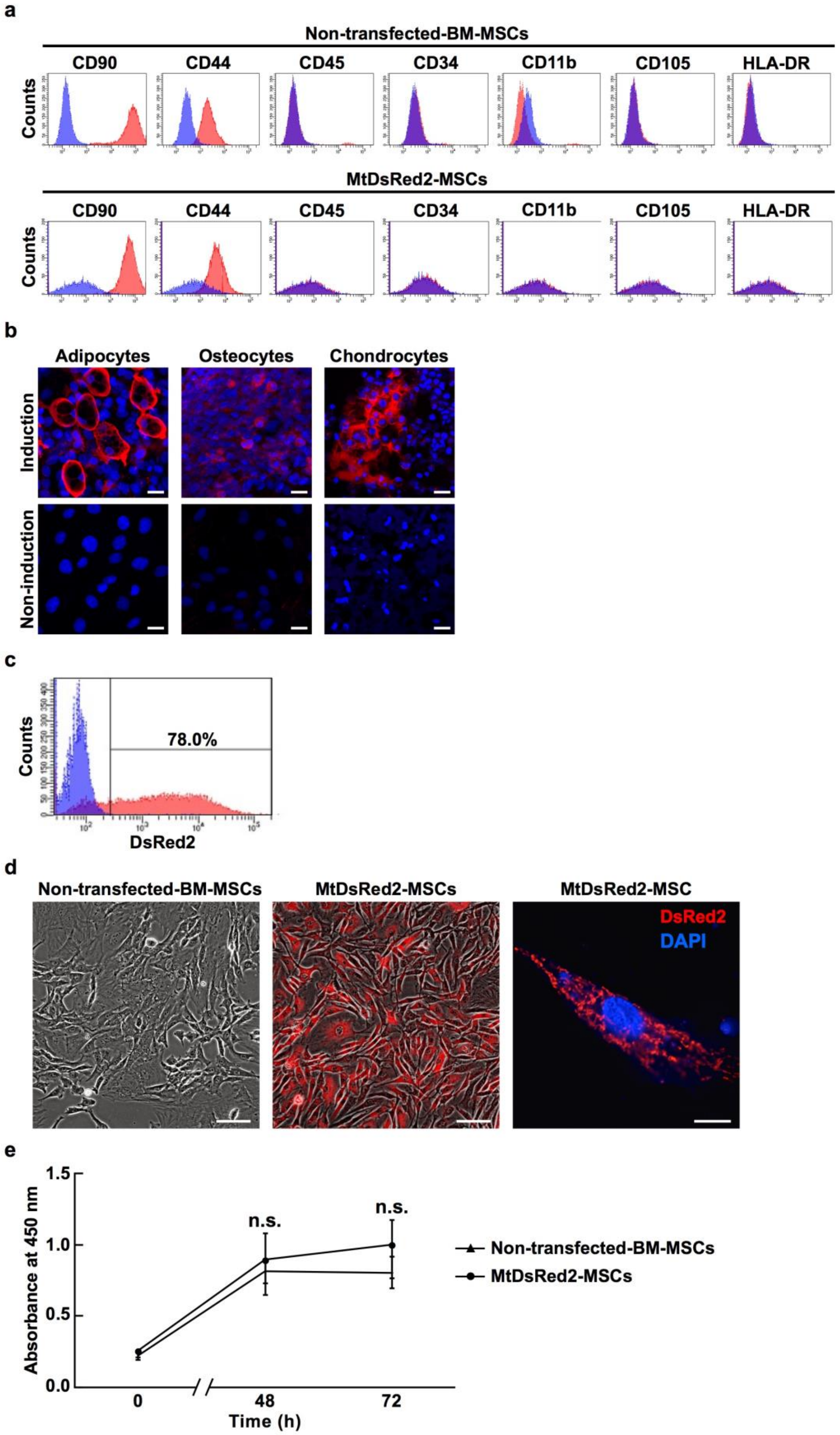

1

Supplementary Figure S2

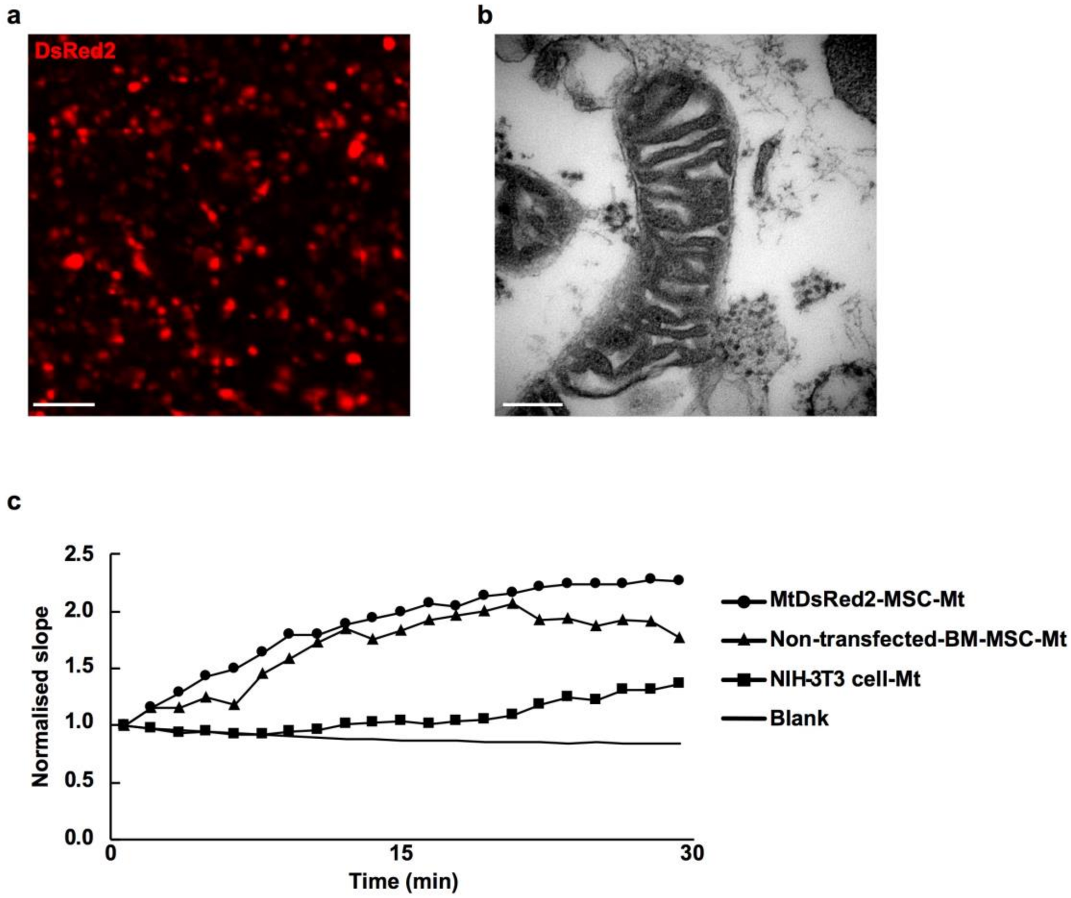

1

Supplementary Figure S3

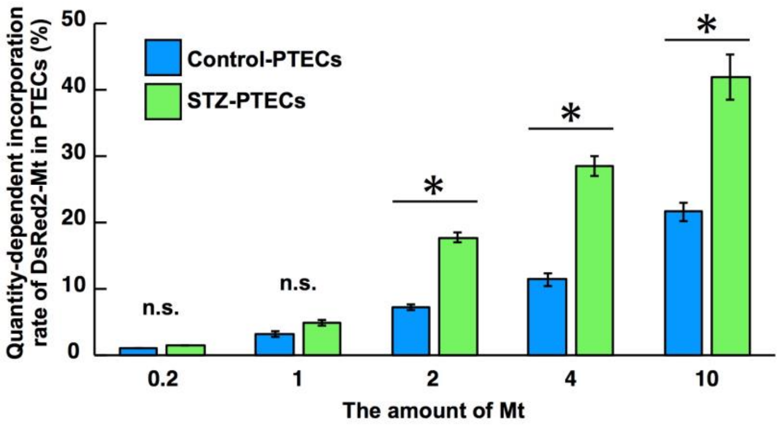

1

Supplementary Figure S4

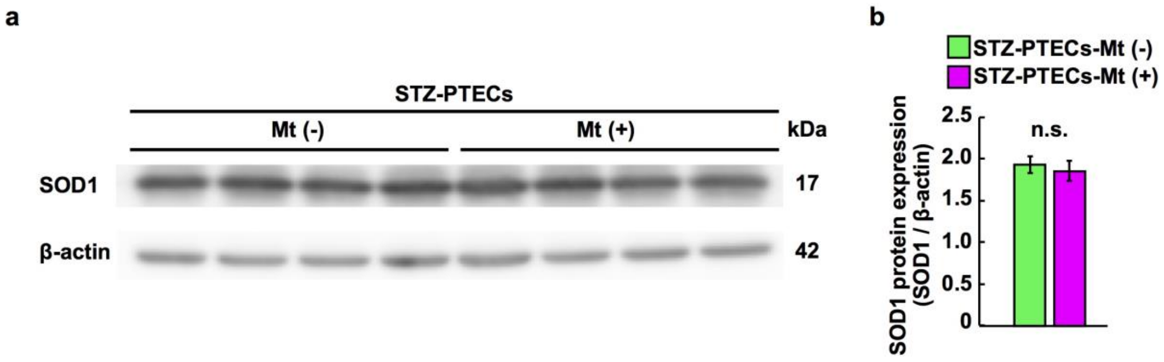

1

Supplementary Figure S5

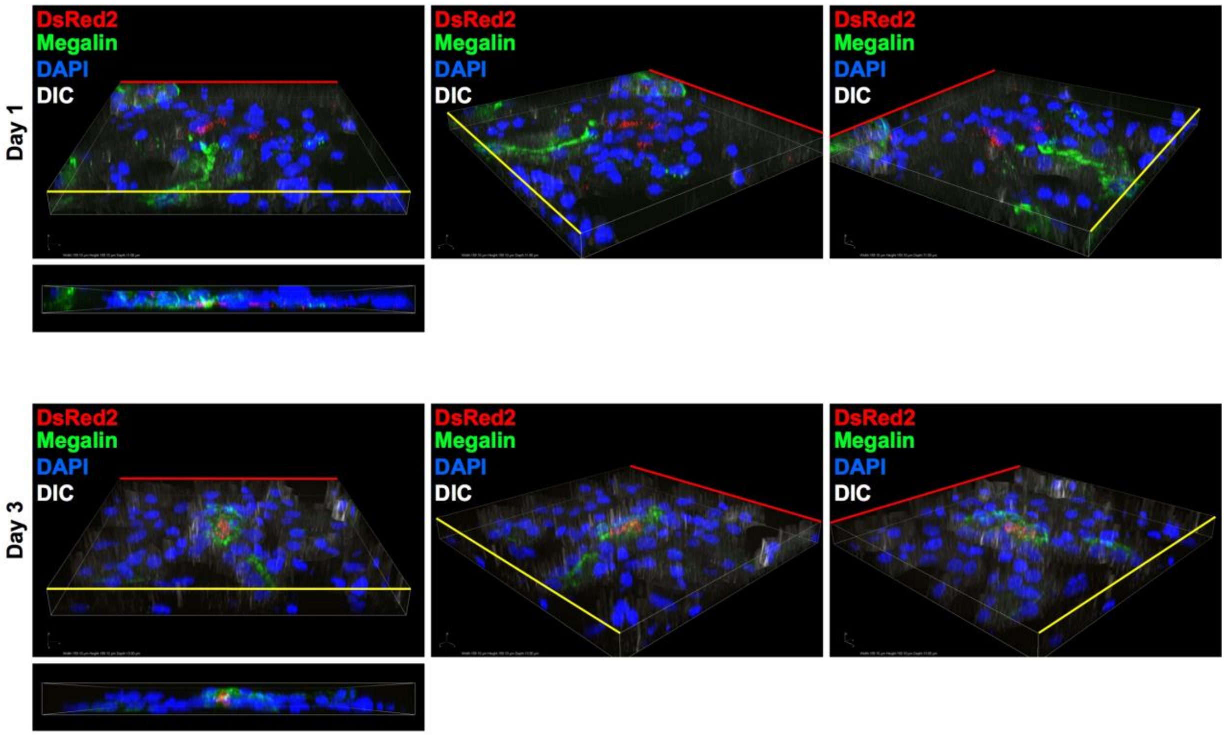

1

Supplementary Figure S6

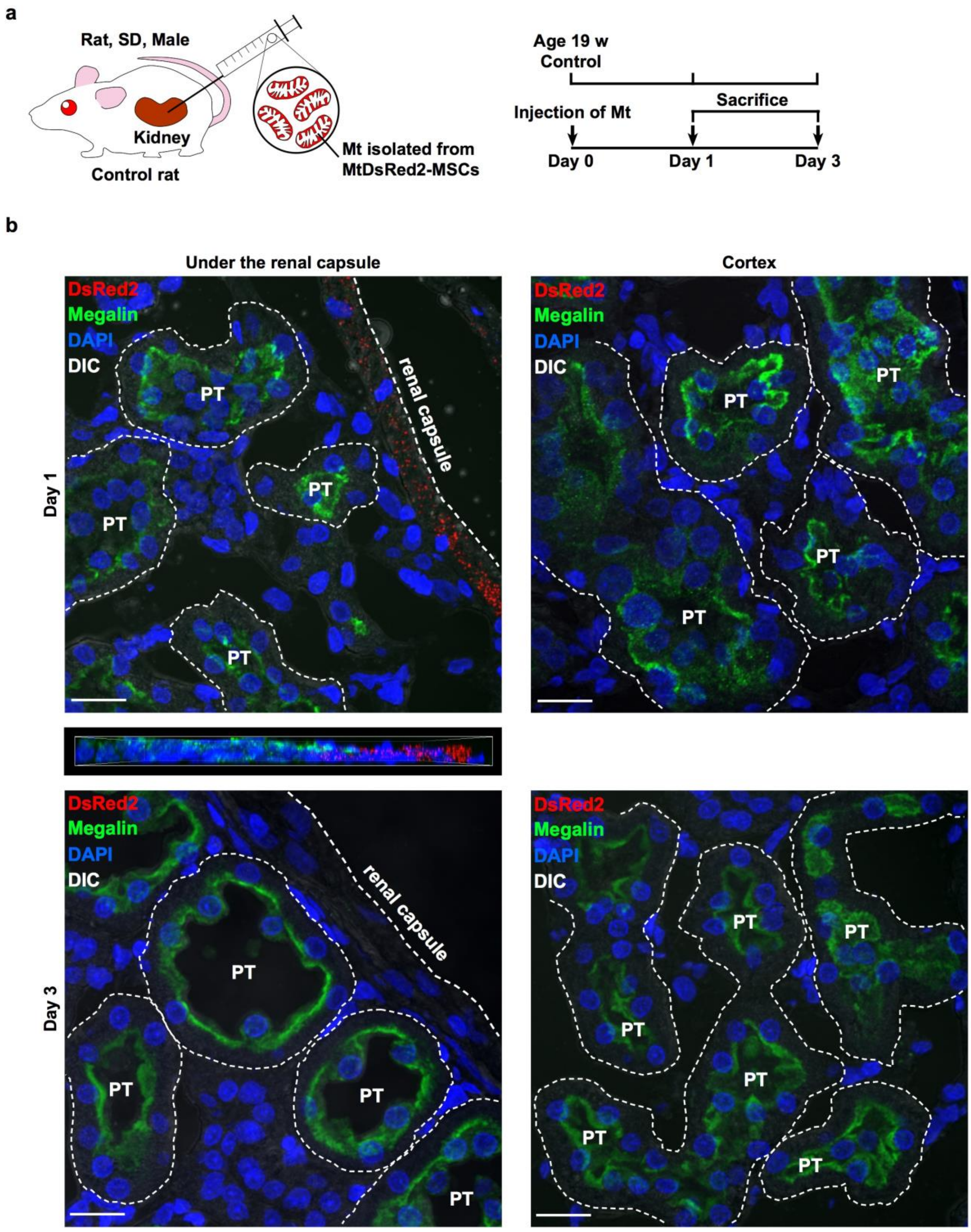

1

Supplementary Figure S7

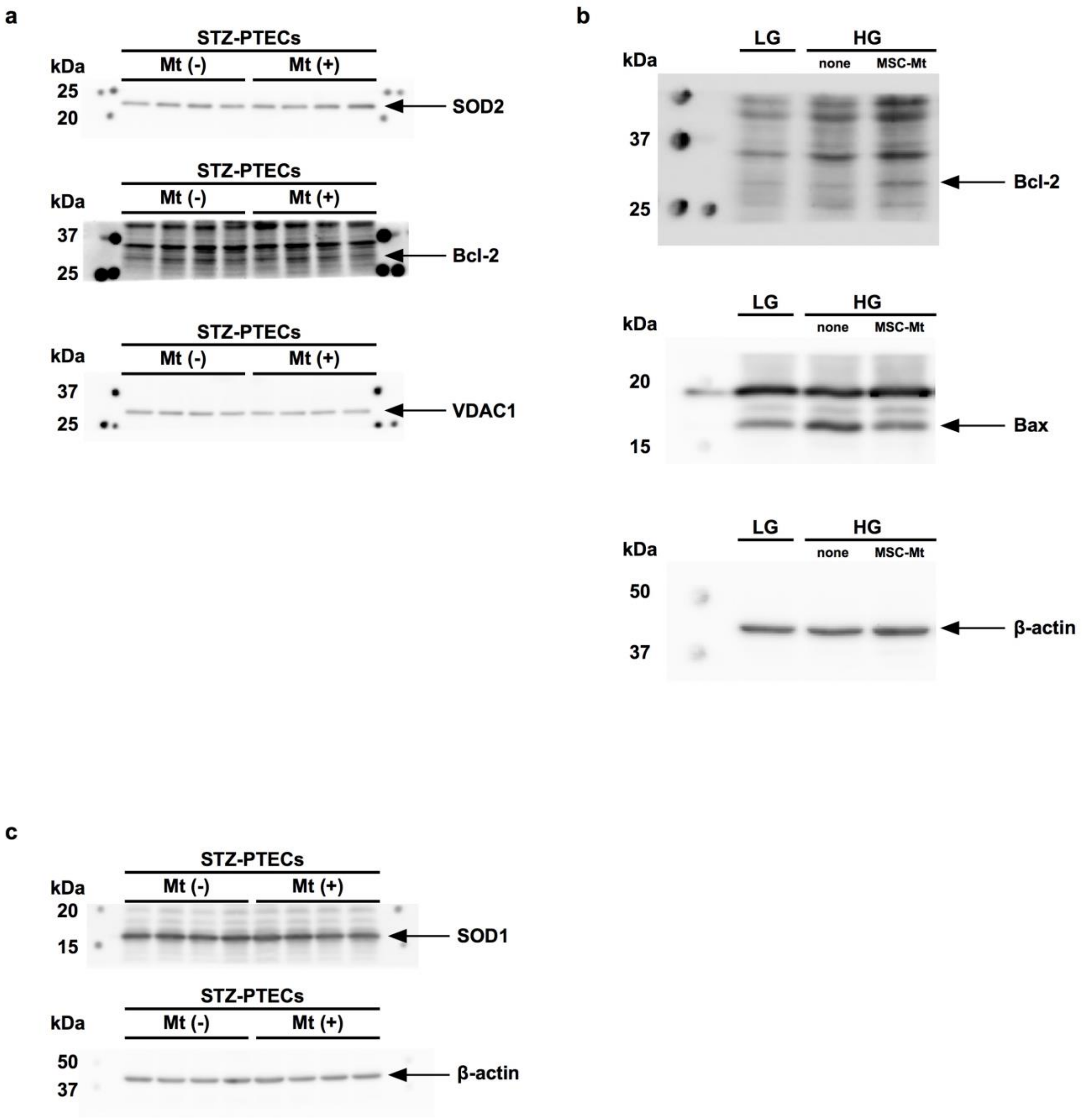

Supplement: Supplementary file 1 — Supplementary information [file 41598_2019_40163_MOESM1_ESM.pdf]
